# CrySyS dataset of CAN traffic logs containing fabrication and masquerade attacks

**DOI:** 10.1038/s41597-023-02716-9

**Published:** 2023-12-15

**Authors:** András Gazdag, Rudolf Ferenc, Levente Buttyán

**Affiliations:** 1 CrySyS Lab, Department of Networked Systems and Services, Budapest University of Technology and Economics, Budapest, Hungary; 2 Department of Software Engineering, University of Szeged, Szeged, Hungary

**Keywords:** Scientific data, Computer science, Electrical and electronic engineering

## Abstract

Despite their known security shortcomings, Controller Area Networks are widely used in modern vehicles. Research in the field has already proposed several solutions to increase the security of CAN networks, such as using anomaly detection methods to identify attacks. Modern anomaly detection procedures typically use machine learning solutions that require a large amount of data to be trained. This paper presents a novel CAN dataset specifically collected and generated to support the development of machine learning based anomaly detection systems. Our dataset contains 26 recordings of benign network traffic, amounting to more than 2.5 hours of traffic. We performed two types of attack on the benign data to create an attacked dataset representing most of the attacks previously proposed in the academic literature. As a novelty, we performed all attacks in two versions, modifying either one or two signals simultaneously. Along with the raw data, we also publish the source code used to generate the attacks to allow easy customization and extension of the dataset.

## Background & Summary

Proof-of-concept demonstrations of attacks have shown the emerging threats against vehicles in recent years. Many attacks exploit the fact that the Controller Area Network (CAN), a widely used network technology in vehicles, lacks security features. As a response, the research community made several propositions to secure the protocol or introduce anomaly detection systems to stop the threats. Recent research has increasingly focused on using machine learning for anomaly detection. A typical property of these approaches is that they require a large dataset for proper model building and evaluation. However, there seems to be a shortage in appropriate datasets that contain a sufficient variety of attacks.

With our dataset, we would like to improve the situation by giving access to a large number of captured CAN logs in various traffic scenarios in both benign and attacked state. Our dataset not only addresses the data quantity requirements of machine learning-based anomaly detection approaches, but we also focus on the peculiarities of the field by capturing traces with different length. The dataset contains shorter traces (with IDs beginning with S-*), which are useful for rapid model development and idea-testing in addition to longer traces (with IDs beginning with T-*) captured in various traffic scenarios for robust real-life evaluation and results. In total, our dataset consists of 1274 CAN traces.

## Methods

**Fig. 1:**
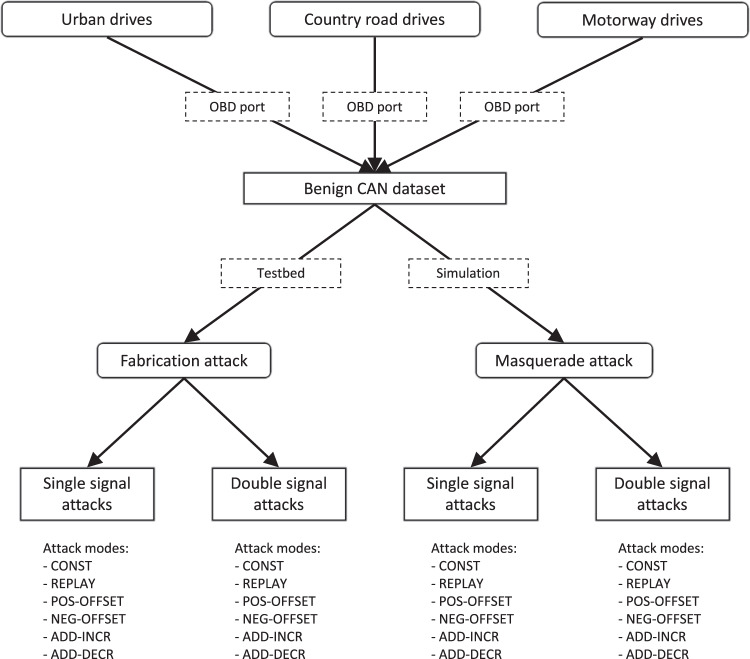
CAN data collection and generation process.

We captured multiple hours of traffic in various traffic scenarios to create a benign dataset. In order to create realistic attacked traces, we chose two approaches to perform attacks. On the one hand, we built a testbed with a physical CAN network to execute attacks affecting the message repetition times. On the other hand, we developed an attack simulator to calculate the effect of timing in different attacks, by modifying only the data part of the CAN messages in the simulator. This hybrid generation approach results in a scalable but still realistic solution. An overview of our data collection and generation process can be seen in Fig. 1.

Besides the previously shown anomaly patterns^1,2^, where the attacker modifies a single signal, we introduce a new modification of the benign signals: double attacks, where the same (or different) attack takes place simultaneously against two CAN signals. Our goal with these anomalies is to test more thoroughly detection systems designed to exploit system-wide communication information, such as signal correlations. We performed all our attacks in single-signal and double-signal modes.

### Benign CAN data captures

The CAN data was captured in our test vehicle through the OBD port. We built a device^3^ to record the raw messages. The captures were performed in a variety of different driving scenarios. The dataset contains 26 recordings: 15 simple maneuver scenarios and 11 complex traffic scenarios, as shown in Table 1. The complex traffic scenarios contain traces captured in an urban environment, on a country road, and during motorway drives.

**Table 1:** CAN trace capture scenarios.

Trace ID	Scenario description	Trace length	Trace size	Number of messages
S-1-1	Driving with about constant 36 km/h speed.	30.08 s	693 KB	17,935
S-1-2	Driving with about constant 36–37 km/h speed.	30.16 s	691 KB	17,888
S-1-3	Driving with about constant 36–37 km/h speed.	30.16 s	695 KB	17,982
S-1-4	Driving with about constant 37 km/h speed.	30.06 s	692 KB	17,911
S-1-5	Driving with about constant 35 km/h speed.	32.72 s	752 KB	19,443
S-1-6	Driving with about constant 37 km/h speed.	29.99 s	672 KB	17,404
S-2-1	Driving with about constant 60 km/h speed.	30.06 s	692 KB	17,909
S-2-2	Driving with about constant 60 km/h speed.	30.01 s	692 KB	17,897
S-2-3	Driving with about constant 59 km/h speed.	30.93 s	713 KB	18,445
S-2-4	Driving with about constant 60 km/h speed.	30.19 s	696 KB	18,000
S-2-5	Driving with about constant 61-62 km/h speed.	31.98 s	738 KB	19,077
S-2-6	Driving with about constant 62 km/h speed.	31.11 s	717 KB	18,553
S-3-1	Speeding up then slowing down from 0 km/h to 50 km/h to 0 km/h.	29.82 s	683 KB	17,669
S-3-2	Speeding up then slowing down from 0 km/h to 40 km/h to 0 km/h.	32.36 s	747 KB	19,327
S-3-3	Speeding up then slowing down from 0 km/h to 40 km/h to 0 km/h.	30.72 s	709 KB	18,335
T-1-1	Driving in urban environment.	430.17 s	10,211 KB	256,921
T-1-2	Driving in urban environment.	1,253.81 s	30,015 KB	748,241
T-1-3	Driving in urban environment.	1,106.71 s	26,433 KB	660,880
T-1-4	Driving in urban environment.	1,576.21 s	37,884 KB	940,154
T-1-5	Driving in urban environment.	1,055.67 s	25,158 KB	629,786
T-1-6	Driving in urban environment.	1,232.86 s	29,431 KB	733,933
T-1-7	Driving in urban environment.	261.73 s	6,189 KB	156,371
T-2-1	Driving on country road.	359.32 s	8,519 KB	214,625
T-2-2	Driving on country road.	371.97 s	8,810 KB	221,907
T-3-1	Driving on motorway.	552.92 s	13,090 KB	328,901
T-3-2	Driving on motorway.	562.09 s	13,333 KB	334,980
	Total:	2 h 33 m 43 s	219,655 KB	5,500,474

The captured data was analyzed to determine the communication properties. The communication contains messages with 18 different CAN IDs. The data fields of the messages were processed with the method proposed by Brent *et al*.^4^ to extract the vehicle signals. We managed to identify and extract 78 signals, which are shown in Table 2.

**Table 2:** Identified CAN signals.

Message ID	Signal Index	Start bit offset	End bit offset
0 × 110	0	6	23
	1	24	39
	2	40	47
	3	48	55
	4	56	63
0 × 120	0	9	19
	1	21	31
	2	34	39
	3	41	51
	4	52	63
0 × 140	0	1	7
	1	14	39
	2	40	63
0 × 180	0	1	12
	1	13	14
	2	15	20
	3	21	28
	4	32	36
	5	37	38
	6	39	47
0 × 1a0	0	12	20
	1	25	31
	2	32	63
0 × 280	0	3	15
	1	19	31
	2	35	47
	3	51	63
0 × 290	0	2	8
	1	18	24
	2	34	40
	3	50	56
	4	57	63
0 × 295	0	6	18
	1	23	31
0 × 300	0	2	3
	1	4	7
	2	8	10
	3	14	25
	4	26	27
	5	28	29
	6	40	55
0 × 301	0	19	47
	1	54	55
0 × 380	0	0	1
	1	2	3
	2	8	11
	3	13	23
	4	32	33
	5	34	35
	6	36	39
	7	45	48
	8	55	56
	9	57	63
0 × 381	0	0	2
	1	3	4
	2	7	15
	3	24	30
	4	31	38
	5	40	47
0 × 383	0	0	4
	1	6	7
	2	10	39
0 × 410	0	9	23
	1	24	32
	2	33	38
	3	39	40
	4	41	48
	5	49	54
0 × 440	0	3	4
	1	5	8
	2	13	22
0 × 4a0	0	16	33
	1	34	47
0 × 510	0	5	15
	1	17	23
	2	25	31
	3	32	63
0 × 531	0	6	39

### Attacks

The inherent insecurity of the CAN bus allows for multiple attacks against vehicles. Taxonomies to categorize these attacks have been proposed in many papers^1,2,5,6^. We describe our performed attacks following the widely used taxonomy of Cho *et al*.^6^.

According to this taxonomy, an attacker can achieve two types of compromise on Electronic Control Units (ECUs): weak and full compromise. A weakly compromised ECU can be used to capture traffic and its normal message transmission can also be suspended (called a suspension attack). In addition to these misdeeds, a fully (or also called strongly) compromised ECU can also inject newly fabricated messages into the CAN bus (called a fabrication or injection attack). In the case of multiple compromised ECUs, if the attacker has weak control over one ECU and full control over another, a new type of attack also becomes possible: masquerade (or modification). In this scenario, the message transmission of the weakly compromised ECU is suspended, and at the same time, a synchronized fabrication attack is also performed using the fully compromised ECU. For the rest of the ECUs on the bus, this attack is transparent from the message repetition time of view: the inter-arrival times of the targeted frames on the bus remain unchanged.

A suspension attack on a weakly compromised ECU has a similar effect on the CAN bus as a device malfunction or failure. As this can happen under benign circumstances as well, safety features are implemented in vehicles to handle such cases without severe consequences. Therefore, our work focused on attacks performed with a fully compromised ECU.

We performed 12 message fabrication and 12 masquerade attacks on our dataset of 26 traces. The attacks have been carried out in both single-signal and double-signal versions. All of the attacks have been performed for two different time durations. The resulting total number of traces in the dataset is 1274 (26 benign and 1248 attacked).

#### Fabrication attack

During a fabrication attack (also called message injection attacks), new messages are injected into the benign traffic. The attacker exploits the fact that ECUs may be implemented so that they accept data at any time. If this is the case, then sending modified CAN frames with a significantly higher frequency can reliably change the behavior of a receiving controller^7,8^. The original and the injected messages appear on the CAN bus simultaneously. The contents of the injected messages can be chosen arbitrarily.

**Fig. 2:**
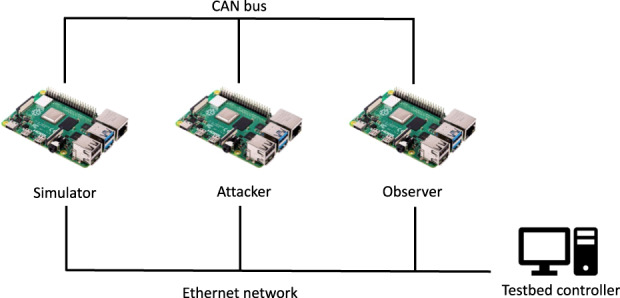
CAN testbed schematics.

We built a CAN testbed from three devices to safely reproduce such an attack in a laboratory environment. In order to remain as close to a real scenario as possible in our testbed, we replayed traffic captured from the test vehicle (with the simulator device) while executing the attacks (with the attacker device). We used a third device (the observer) to capture the effects of the attack on the replayed traffic. The schematic of the testbed is shown in Fig. 2.

#### Masquerade attack

A masquerade attack hl(also called message modification attacks) is the most complicated to be performed on an actual vehicle because two ECUs have to be differently compromised in a coordinated way. This attack is also the most stealthy option for an attacker, as there are no additional messages on the CAN bus, and the timing of the normal packets remain unchanged. This property makes this attack easy to simulate: we modified the data contents of some messages of our benign capture logs, leaving all other aspects of the capture unchanged to achieve the effect of a masquerade attack. Overall, we performed the same number and type of attacks in the masquerade cases as during the fabrication attacks.

#### Signal modification strategies

**Fig. 3:**
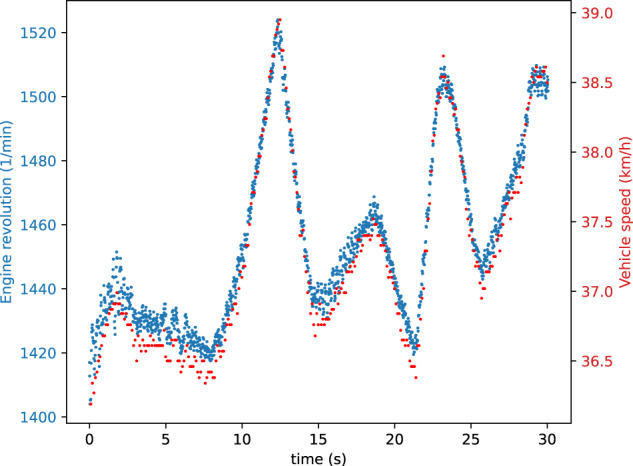
Example benign CAN signal (S-1-4).

We chose two signals as the target of our tests: the vehicle speed and the engine revolution signals (Fig. 3). We found these signals in the CAN communication using manual reverse engineering steps and validated our finding with the method presented by Lestyán *et al*.^9^.

**Fig. 4:**
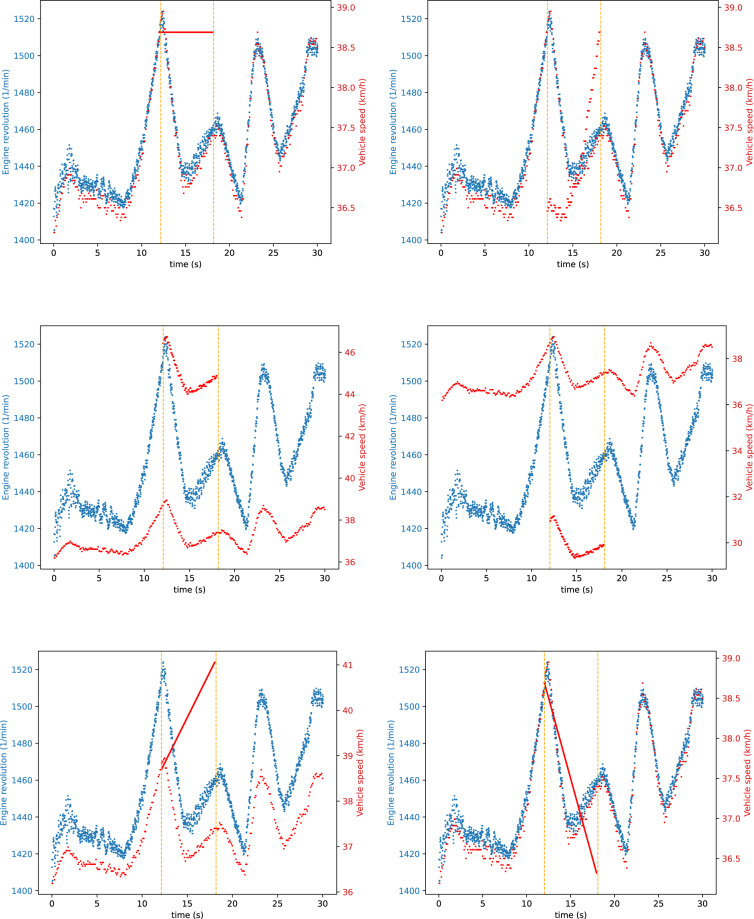
Single signal injection attacks (S-1-4). Used signal modification strategies: CONST, REPLAY, POS-OFFSET, NEG-OFFSET, ADD-INCR, ADD-DECR.

**Fig. 5:**
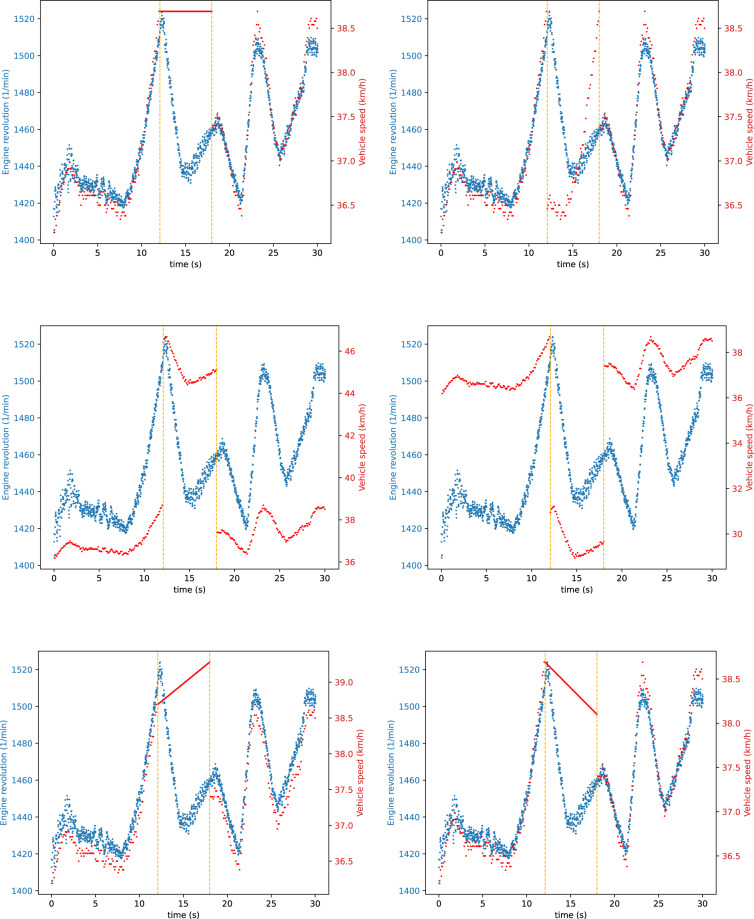
Single signal modification attacks (S-1-4). Used signal modification strategies: CONST, REPLAY, POS-OFFSET, NEG-OFFSET, ADD-INCR, ADD-DECR.

**Fig. 6:**
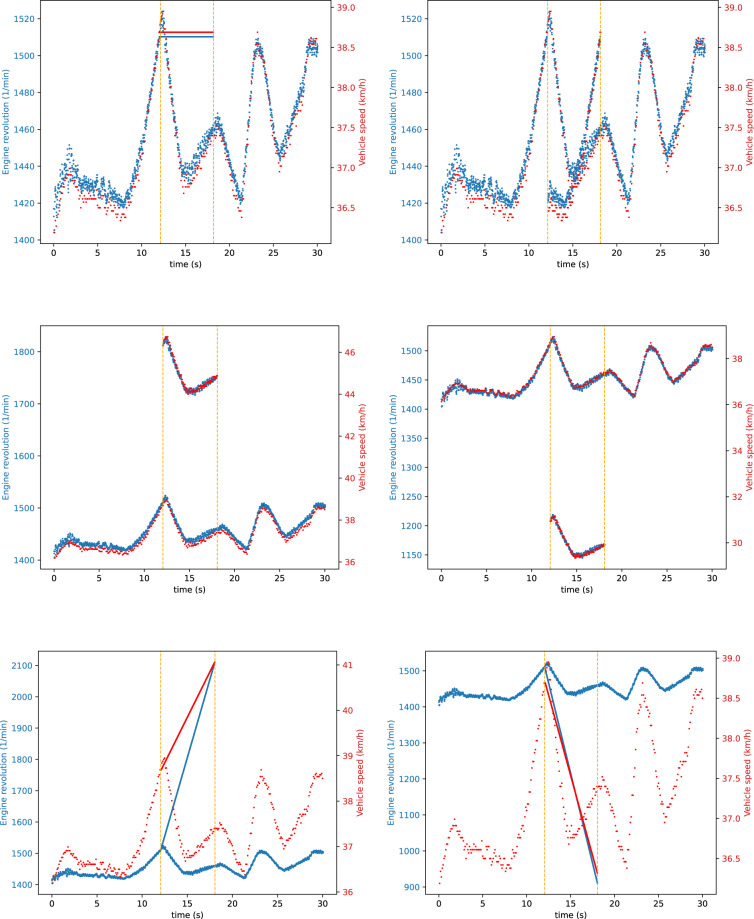
Double signal injection attacks (S-1-4). Used signal modification strategies: CONST, REPLAY, POS-OFFSET, NEG-OFFSET, ADD-INCR, ADD-DECR.

**Fig. 7:**
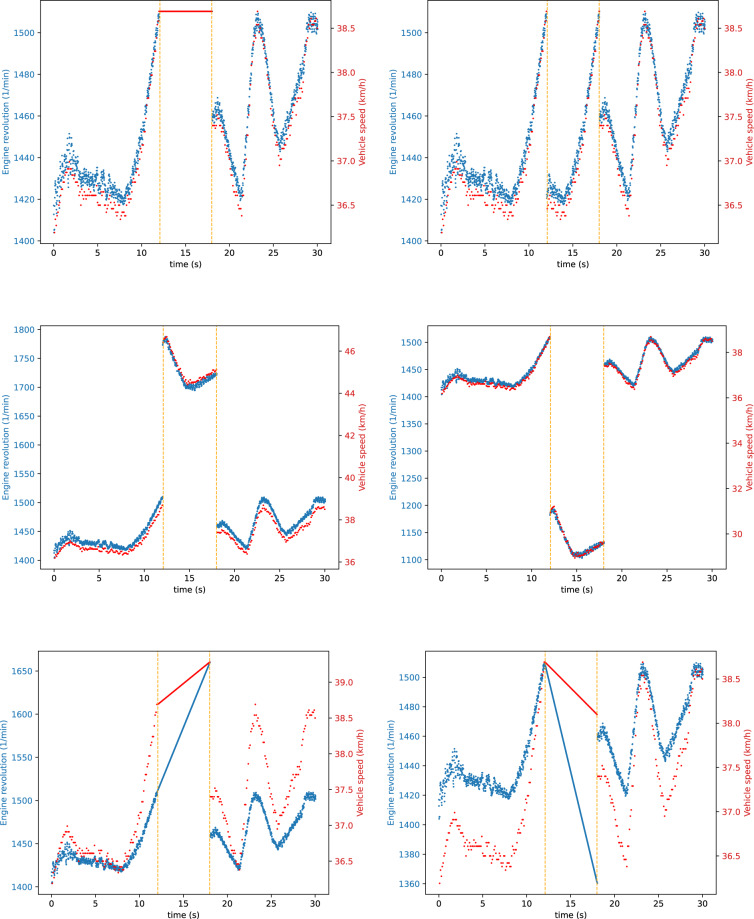
Double signal modification attacks (S-1-4). Used signal modification strategies: CONST, REPLAY, POS-OFFSET, NEG-OFFSET, ADD-INCR, ADD-DECR.

We defined six signal modification strategies that we performed during both the fabrication and the masquerade attacks. Furthermore, we executed the same attacks once only on one signal (Figs. 4, 5), then targeting two signals simultaneously (Figs. 6, 7). This wide range of attacks cover many strategies, allowing for a thorough evaluation of defense mechanisms. The chosen signal modification strategies are the following:

- CONST: The attacker replaces the CAN signal values with a constant in every message.
- REPLAY: The attacker replaces a CAN signal value with a previously captured value from the traffic. This attack takes twice as long compared to the others: first, the attacker records the signal values, then in the second half of the attack, it replays them.
- POS-OFFSET: The attacker adds a constant value to the CAN signal in each message.
- NEG-OFFSET: The attacker adds a constant value to the CAN signal in each message.
- ADD-INCR: The attacker adds a continuously incrementing value to the CAN signal in each message. This causes a slow but growing shift away from the original value.
- ADD-DECR: The attacker subtracts a continuously decrementing value in each message from the CAN signal. This causes a slow but growing shift away from the original value.

## Data Records

The dataset is available at Figshare^10^. Multiple files belong to each test case. The files containing the benign test cases are the following:

- *[TraceID]-benign.log*: CAN trace file with the raw messages.
- *[TraceID]-benign.json*: metadata about the trace (e.g. capture details).
- *[TraceID]-benign-speedAndRevolutionSignal.pdf*: plot of the speed and engine revolution signals.

The files containing the attacked cases are organized in the following way:

- *[TraceID]-malicious-[Attack-type].log*: CAN trace file with the raw messages.
- *[TraceID]-malicious-[Attack-type]-inj-messages.log*: in case of a message injection attack, the injected messages are stored separately as well.
- *[TraceID]-malicious-[Attack-type].json*: metadata about the trace (e.g. capture details and trace file information).
- *[TraceID]-malicious-[Attack-type]-speedAndRevolutionSignal.pdf*: plot of the speed and revolution signals in two format.
- The TraceIDs for each of our scenarios can be found in Table 1. The structure of a raw CAN trace follows the format used by the SocketCAN Linux package.

## Technical Validation

### CAN data recorder validation

We captured and transmitted CAN packets with a Raspberry Pi based recorder. Using the PiCAN2 board from SK Pand, we were able to handle messages up to 1 Mbps speeds, the maximal transmission speed of the CAN bus. We verified with measurements, using commercial tools, that our device processes each CAN frame without packet loss.

We executed “dry runs” of our testbed. During these executions, we replayed messages with the simulator device and recaptured them with the observer device without an attacker’s intervention. These tests validated that all messages arrive in our testbed, and the inter-arrival times between messages remain unchanged.

### Attack validations

Our attacks target two CAN signals: (i) the engine revolution signal and (ii) the vehicle speed signal. Both signals are displayed on the dashboard; thus, the effects of the attacks have been manually validated first to show that they have an actual impact on the vehicle.

#### Fabrication attack validation

We tested our testbed for the correctness of the fabrication attacks in two ways. First, we performed the testbed validation tests for every measurement to detect potential message loss. Second, we plotted the resulting signal after the attacks to verify the achieved effect visually. The result was rejected if any inconsistency was found, and the test re-executed. If the result passed all the checks, an automated visualization and documentation of the test case was executed.

#### Masquerade attack validation

The execution of these attacks only modifies the data part of the messages. Therefore, we only had to check that the modifications were aligned with our signal modification goals. Similarly to fabrications attacks, we plotted the resulting signals and validated that the behavior of the modified signal matches the goal.

## Usage Notes

### Dataset customization

We release the source code used for the attack generation along with the dataset. The targeted signals and attack concepts have been verified; therefore, our code can generate further attacks. This approach significantly extends the potential size of the dataset.

### Comparison to other datasets

The lack of available datasets has significantly hindered the research on CAN security^11^. Capturing real data and performing attacks require a significant effort and special expertise in the automotive field. Therefore, datasets with a wide range of attacks are required for advancements in the field.

Previous datasets primarily focus on fabrication attacks due to the relatively easy execution of these attacks. Although the significance of a fabrication attack has been shown in successful vehicle compromises, the drastic changes of these attacks in the frame repetition times allow the development of effective detection methods. Masquerade attacks are more powerful attack methods. Therefore, detection algorithms should also be tested against those. Currently available datasets either lack some of the desired features of the attacks or the attack circumstances are artificial. A summary of the datasets is shown in Table 3.

**Table 3:** CAN Dataset comparison.

Dataset	Year	Data labeled	Number of different attack strategies	Fabrication attack	Masquerade attack
CAN Dataset for intrusion detection (OTIDS)	2017	—	3	Real	Simulated
Car-Hacking Datase	2018	✓	3	Real	Simulated
Automotive CAN Bus Intrusion Dataset v2	2019	✓	5	Real and simulated	Simulated
SynCAN	2019	✓	5	Simulated	Simulated
ROAD	2020	—	7	Real	Simulated
**CrySyS dataset**	**2023**	✓	**12**	**Real**	**Simulated**

The HCRL Lab released two CAN datasets with different attacks called “CAN Dataset for intrusion detection (OTIDS)”^12^ and “Car-Hacking Dataset”^13^. Both datasets contain only fabrication attacks achieving different goals like, DoS, fuzzing, spoofing, or impersonation attacks.

The “Automotive Controller Area Network (CAN) Bus Intrusion Dataset v2”^14^ dataset contains three different types of attacks: suspension, fabrication, and masquerade attacks. Their goal during the fabrication attacks is to perform a DoS, fuzzing, or replay attack. During a masquerade attack, they replace the frame data bytes with an FF value. Although this is a new type of attack, detecting this significant change is a manageable task.

The SynCAN (Synthetic CAN Bus Data) dataset^15^ contains only extracted CAN signals instead of the original CAN frames. The attacks are synthetically generated and their impact is unknown. The attack generation tactics have a similar approach to that of ours (e.g. they also perform a CONST attack called Plateau, an ADD-INCR attack called Continous Change, and a REPLAY attack called Playback), but the dataset is significantly smaller compared to ours.

The ROAD dataset^11^ can be considered the most complete dataset so far. It contains both fabrication and masquerade attacks that are physically verified to have an impact on the vehicle. Although their tests were performed on a real vehicle and not on a testbed, they executed their experiments on a dynamometer to remain safe during the test. This approach ensures that the attacks are executed on an existing CAN network; however, the vehicle is in a test environment during the execution. Therefore any external circumstance caused by a real environment (e.g. traffic scenarios) is missing from their data.

There are further CAN datasets available for purposes other than attack detection (see e.g., the “Automotive CAN bus data: An Example Dataset from the AEGIS Big Data Project”). As their contents are unusable for our research goals, we excluded them from the comparison.

## Data Availability

The source code used for the dataset generation is open source (https://github.com/CrySyS/CAN-Dataset-Generator), which allows others to extend or modify the dataset. Fabrication attack generation requires a few easily accessible hardware components, while the masquerade attacks can be generated on any general-purpose computer.
